# Prevalence of overweight/obesity, and associated factors among adolescents aged 12 ∼ 15 in Shandong Province, China: A cross-sectional study

**DOI:** 10.1016/j.pmedr.2024.102831

**Published:** 2024-07-22

**Authors:** Zhihao Huang, Zhiqi Tian, Jian Cui, Guan Wang, Jiyan Chen

**Affiliations:** a School of Big Data and Fundamental Sciences, Shandong Institute of Petroleum and Chemical Technology, Dongying, China; b Department of Clinical Laboratory, Shengli Oilfield Central Hospital, China

**Keywords:** Overweight/obesity, Adolescents, Univariable chi-square test, Multivariable logistic regression analysis

## Abstract

Overweight/obesity among adolescents in Shandong Province, China, has been rising, posing significant public health challenge. Comprehensive investigation is needed to develop effective interventions. Following the Strengthening the Reporting of Observational Studies in Epidemiology guidelines, a stratified random cluster sampling approach was used from September to October 2023 across 17 cities in Shandong Province. The study included 165 middle schools, surveying 99,638 students aged 12 ∼ 15. After applying exclusion criteria, 97,356 students (97.71% effective rate) completed anthropometric measurements and questionnaires. Overweight/obesity were assessed based on national and international standards. Univariable chi-square test and multivariable logistic regression were used to analyze factors influencing overweight/obesity. In 2023, the overweight/obesity rate among 12 ∼ 15-year-olds in Shandong was 19.75%. Significant factors included sex, age, residence, family income, parental weight status and activity, mother’s gestational diabetes history, birth weight, physical activity, sleep, screen time, homework, and diet. Girls, older adolescents, and those with physically active parents or who themselves engaged in over 1.5 h of daily physical activity had lower odds of being overweight/obese. Adequate sleep and frequent consumption of vegetable and fruit were also protective. Higher odds were associated with urban residence, high family income, overweight/obese parents, maternal gestational diabetes, high birth weight, excessive screen time, extensive homework, and frequent fast food consumption. Overweight/obesity in Shandong adolescents is influenced by multiple determinants. Holistic interventions addressing genetic, behavioral, and environmental factors are essential for promoting healthier lifestyles and reducing the prevalence in this demographic.

## 1 Background

In the modern global landscape, accelerated socio-economic progression has profoundly transformed people's lifestyles, paralleled by the ubiquitous integration of technological gadgets and modern transportation. While devices like smartphones and electronic appliances, coupled with advanced transportation methods, undeniably enhance our daily conveniences, they introduce consequential downsides. Foremost among these is the burgeoning challenge of overweight/obesity, particularly poignant during the formative adolescent years. Across the world, irrespective of developmental status, obesity during childhood and adolescence is increasingly recognized as a paramount public health issue (Bagnall et al., 2019). In recent years, the trend of obesity among children and adolescents worldwide has been sharply on the rise (Han et al., 2010). The global prevalence of obesity has increased substantially over the past 40 years, from less than 1% in 1975, to 6 ∼ 8% in 2016, among girls and boys (Jaacks et al., 2019). The statistical analysis from 1980 to 2015 conducted by the Global Burden of Disease 2015 Obesity Collaborators across 195 countries unveiled a concerning reality: there were 107.7 million children suffering from obesity, and in some countries, its growth rate has surpassed that of the adult population (Afshin et al., 2017). Notably, in the United States, between 2011 and 2012, the obesity prevalence among adolescents aged 2 ∼ 19 was 17.7%, a figure that rose to 21.5% between 2017 and 2020 (Hu and Staiano, 2022). Significantly, rapid economic development in Asian regions, leading to notable shifts in nutrition and physical activity behaviors, has further magnified the issue of childhood and adolescent obesity (Tham et al., 2023). Presently, the obesity rates among children and adolescents in many developing countries are also witnessing a swift ascent (Kelishadi, 2007).

China is currently at a pivotal juncture in its economic transition, with profound changes occurring in the lifestyles, living environments, and health awareness of its citizens. The health challenges triggered during this phase warrant our utmost attention. Adolescence is a crucial period for an individual's physical and mental health development. At this juncture, the health status of adolescents not only profoundly impacts the well-being of individuals and their families but also bears significance to the overall health quality and talent development of the Chinese nation. However, the present health conditions of middle and primary school students in China are causes for reflection. Among the most conspicuous issues is the sharp rise in the detection rate of overweight/obesity. Statistics indicated that in China, the prevalence of overweight/obesity among children and adolescents aged 7 ∼ 17 was steadily climbing (Dong et al., 2019). From 1995 to 2014, the overweight rate surged from 3.8% to 14.3%, while the obesity rate also increased from 0.6% to 4.1% (Dong et al., 2018). The outbreak of the Corona Virus Disease 2019 pandemic had a significant impact on obesity rate (Clemmensen et al., 2020). Reports suggested that during this pandemic, the prevalence of obesity among Chinese adolescents showed an upward trend (He et al., 2022, Long et al., 2023, Wen et al., 2021, Yang et al., 2020, Yang et al., 2022). This phenomenon foreshadowed a grave threat to the physical health of children and adolescents in the country. Additionally, research indicated that children and adolescents in urban and economically developed areas had significantly higher rate of overweight/obesity compared to their counterparts in rural and economically underdeveloped areas (Musa et al., 2016). Even more concerning is the fact that the obesity rate in rural areas is also on a comprehensive rise (Zhang et al., 2016), further attesting that the issue of overweight/obesity among children and adolescents in China has entered a new and even more severe epidemic phase.

Numerous scientific studies continued to reveal that obesity during childhood was becoming an urgent and significant health concern that could not be overlooked. Childhood obesity not only significantly increases the risk of various chronic diseases during adolescence, including pediatric asthma (Green, 2014), diabetes (Frayling et al., 2007), and cardiovascular diseases (Bridger, 2009), but even more worrisome, it may also elevate the risk of developing cancer in the future (Weihrauch-Blüher et al., 2019). Furthermore, obesity has been identified as a risk factor for metabolic syndrome in adulthood (Lloyd et al., 2012). If not properly managed and treated, obesity can also induce premature dysregulation of the immune system (Fang et al., 2020) and non-alcoholic fatty liver disease (Widhalm and Ghods, 2010), among other health issues. A prospective study observed 1,309 participants and found that adolescents with higher BMI values had a greater risk of becoming obese in adulthood (Wang et al., 2008). However, the challenges obesity presents to children extend beyond just physiological health. In fact, its ramifications deeply permeate their academic, psychological, and social lives. Numerous studies have shown that, due to weight-related issues, obese children often become targets in their communities, facing discrimination, ridicule, and even more severe forms of bullying (Koyanagi et al., 2020, Rupp and McCoy, 2019, Kukaswadia et al., 2011, Jansen et al., 2014). These psychological stresses and negative emotional experiences not only severely undermine their self-esteem and confidence but can also lead to depression, anxiety, and a host of other mental disorders (Rao et al., 2020, Chao et al., 2019, Sheinbein et al., 2019). Undoubtedly, these repercussions can further impact their academic performance and daily life negatively. Therefore, addressing the issue of overweight/obesity is not just about safeguarding the physiological well-being of children but also about ensuring their mental health and the quality of their future lives.

In summary, it is evident that both within China and across various regions globally, we are confronted with an escalating public health challenge: overweight/obesity among children and adolescents. At such a pivotal historical juncture, it is of paramount importance to intensify research and preventive measures addressing this issue, ensuring the health of this unique demographic, and laying a solid foundation for the future health and prosperity of both China and the world at large. Prioritizing the health of our young population aligns with the third goal of the United Nations' Sustainable Development Goals for 2030, which advocates for ensuring healthy lives and promoting wellbeing for all at all ages (Huang and Chang, 2022). This study aimed to analyze data related to the physical fitness and health survey of students aged 12 ∼ 15 in Shandong Province in 2023. The intent was to understand the current overweight/obesity situation of students in this age bracket in Shandong Province and to explore the underlying factors, thus providing a basis for the holistic health development of our youth.

## 2 Materials and methods

### 2.1 Design and participants

This study was conducted in accordance with Strengthening the Reporting of Observational Studies in Epidemiology guidelines (von Elm et al., 2007). Using a stratified random cluster sampling method, students from 165 middle schools (aged 12 ∼ 15) in 17 cities of Shandong Province were selected as research subjects between September and October 2023. Students were stratified by grade and whole classes were randomly sampled as units, with a total of 99,638 individuals surveyed. Among the surveyed students, we excluded those with significant organ diseases, physical disabilities or deformities. Of those surveyed, 97,356 individuals completed the height and weight measurements and questionnaire, yielding a validity rate of 97.71 %. Among the valid surveys, males accounted for 50.02 % (48,700 individuals), and females accounted for 49.98 % (48,656 individuals). The distribution by age was as follows: 20.82 % (20,269 individuals) were 12 years old, 24.80 % (24,144 individuals) were 13 years old, 25.46 % (24,784 individuals) were 14 years old, and 28.92 % (28,159 individuals) were 15 years old. The average age was 13.62 ± 1.109 years.

### 2.2 Measurement of overweight/obesity and questionnaire survey

All participants and investigators in this study underwent standardized training, ensuring accuracy in height and weight assessments and familiarity with field epidemiological techniques. Before the survey commencement, students were informed about the study's purpose and potential implications. Subsequent to the height and weigh measurements, students received questionnaires and were allotted a day to consult their parents and complete them. Upon collection, the questionnaires were systematically organized, with data promptly entered for comprehensive analysis. This study was approved by the Ethics Committee of Shandong Institute of Petroleum and Chemical Technology (registered number: KY-2023-018). All methods were carried out in accordance with relevant guidelines and regulations. Informed consent was obtained from all participants involved in the study.

Based on the National student physical health standard (Revised 2014) (Zhai et al., 2022), it was mandated to measure students' height and weight using a uniform medical-grade scale. A designated individual ensured the calibration of these instruments. The personnel conducting the measurements were all trained health professionals who adhered to standard measurement procedures. When being measured, students were advised to empty their bladder, remove shoes, hats, outer garments, and head accessories. They should stand upright at the center of the scale platform, arms hanging naturally and eyes looking forward. Measurements were taken three times in succession, with the average value recorded. The height was accurate to 0.1 cm, and the weight to 0.1 kg.

Body mass index was calculated as weight (kilogram) divided by height (meter) squared. The international age-and sex-specific child cutoff points of the International Obesity Task Force (Cole et al., 2000) were used to define overweight/obesity.

The questionnaire section was conducted anonymously, with the survey content specifically including: Demographic characteristics: sex, age, place of residence, family annual income (yuan), father’s education level, mother’s education level, weight status of parents, physical activity of parents, mother’s history of gestational diabetes mellitus (GDM), birth weight; Daily life behaviors: physical activity duration daily (hour), sleep duration daily (hour), screen duration daily (hour), homework duration daily (hour), times of meat intake weekly, times of vegetable intake weekly, times of fruit intake weekly, times of eggs intake weekly, times of milk intake weekly, times of fast food consumption weekly.

### 2.3 Statistical analysis

Continuous data were described as means and standard deviations, and categorical data were described as the number of cases and percentages (%). In the univariable analysis, the chi-squared (χ^2^) test was used. Variables with statistically significant differences (P<0.05) from the univariable analysis were incorporated into the multivariable logistic regression analysis (Bzovsky et al., 2022). The subsequent stage of analysis utilized logistic regression, systematically yielding results in the form of odds ratio (OR) accompanied by 95 % confidence interval (CI) for precise delineation of estimation accuracy. To affirm the integrity of our findings, a detailed evaluation of the logistic regression model's fit was executed, guided by the Hosmer-Lemeshow test. This diligent process not only validated the model's accuracy but also fortified the robustness of our analytical framework, rooted in the tenets of statistical thoroughness and accuracy. A database was established using EpiData version 3.1 (EpiData Association), and data analysis was conducted using Statistical Product and Service Solutions version 27.0 (International Business Machines Corporation).

## 3 Results

### 3.1 The epidemiological characteristics of overweight/obesity rate among adolescents in Shandong Province

According to the overweight/obesity data surveyed in 2023 for adolescents aged 12∼15 in Shandong Province, the overall overweight/obesity rate was 19.75 %. When broken down by sex, the rate was slightly higher in boys at 20.55 %, compared to 18.95 % in girls, with the difference being statistically significant (P<0.05). Interestingly, age played a role. The 12-year-olds had the highest rate of overweight/obesity at 20.58 %, followed by 13-year-olds at 19.87 %, 14-year-olds at 19.55 %, and 15-year-olds at 19.21 %. This variation across age groups was statistically significant (P<0.05). Place of residence was another influential factor. Adolescents from urban settings had a higher overweight/obesity rate (20.07 %) compared to their rural counterparts (19.17 %), with the difference being statistically significant (P<0.05). Economic disparities, as evidenced by family annual income in yuan, also held considerable sway over overweight/obesity rates in adolescents. For families earning ≤ 100,000, the rate was 19.52 %. Those with incomes between 100,001 and 200,000 had a rate of 19.61 %. The rate slightly increased to 20.18 % for incomes ranging from 200,001 to 300,000. Notably, families with an income of ≥ 300,000 exhibited the highest prevalence at 20.80 %. These variations across income tiers were statistically significant (P<0.05). Weight status of parents also played a part in the children's overweight/obesity rates. The rate stood at 18.54 % when neither parent was overweight/obese. This number rose to 22.23 % if one parent was affected and further increased to 25.08 % if both parents were overweight/obese. This variation was statistically significant (P<0.05). Furthermore, the physical activity of parents impacted the children's rates. The overweight/obesity rate was highest at 20.98 % when neither parent was active. This rate decreased to 19.57 % when one parent was active and further reduced to 16.80 % if both parents were active, showing a significant trend (P<0.05). Additionally, mother’s history with Gestational Diabetes Mellitus (GDM) was a noteworthy factor. Adolescents whose mothers had a history of GDM had a significantly higher overweight/obesity rate of 26.92 %, compared to 19.59 % for those whose mothers did not have such a history (P<0.05). Lastly, birth weight played a role. Adolescents with a normal birth weight had a rate of 19.62 %, compared to 19.49 % for those with a low birth weight, and 22.06 % for those with a high birth weight. Again, these variations were statistically significant (P<0.05) (Table 1).

**Table 1:** Overweight/obesity prevalence according to demographic characteristics of participants in Shandong Province, 2023.

Demographic characteristics	Normal weight(%)	Overweight/obesity(%)	χ^2^	*P*
Sex			39.564	0.000
Male	38,692(79.45)	10,008(20.55)		
Female	39,438(81.05)	9,218(18.95)		
Age (year)			14.850	0.002
12	16,097(79.42)	4,172(20.58)		
13	19,346(80.13)	4,798(19.87)		
14	19,938(80.45)	4,846(19.55)		
15	22,749(80.79)	5,410(19.21)		
Place of residence			11.491	0.001
Rural	28,205(80.83)	6689(19.17)		
Urban	49,925(79.93)	12,537(20.07)		
Family annual income (yuan)			8.561	0.036
≤ 100,000	26,709(80.48)	6,477(19.52)		
100,001 ∼ 200,000	35,154(80.39)	8,576(19.61)		
200,001 ∼ 300,000	10,064(79.82)	2,544(20.18)		
≥ 300,000	6,203(79.20)	1,629(20.80)		
Father’s education level			1.657	0.437
Junior high school or below	14,413(80.58)	3,473(19.42)		
Senior high school	23,013(80.25)	5,664(19.75)		
Above senior high school	40,704(80.14)	10,089(19.86)		
Mother’s education level			3.412	0.182
Junior high school or below	16,277(79.98)	4,074(20.02)		
Senior high school	21,201(80.03)	5,291(19.97)		
Above senior high school	40,652(80.48)	9,861(19.52)		
Weight status of parents			254.210	0.000
Neither parent overweight/obesity	56,788(81.46)	12,928(18.54)		
One parent overweight/obesity	16,832(77.77)	4,811(22.23)		
Both parents overweight/obesity	4,441(74.92)	1,487(25.08)		
Physical activity of parents			162.006	0.000
Neither parent active	41,197(79.02)	10,938(20.98)		
One parent active	20,019(80.43)	4,872(19.57)		
Both parents active	16,914(83.20)	3,415(16.80)		
Mother’s history of GDM			68.649	0.000
No	76,618(80.41)	18,668(19.59)		
Yes	1,512(73.08)	557(26.92)		
Birth weight			20.266	0.000
Normal birth weight	67,820(80.38)	16,549(19.62)		
Low birth weight	5,911(80.51)	1,431(19.49)		
High birth weight	4,399(77.94)	1,245(22.06)		

Overweight/obesity prevalence according to demographic characteristics of participants in Shandong Province, 2023.

### 3.2 Univariable analysis of overweight/obesity rate in adolescents from Shandong Province

The univariable analysis delineated that several factors were inextricably linked with the overweight/obesity rate among adolescents aged 12 ∼ 15. These encompassed physical activity duration daily (hour), sleep duration daily (hour), screen duration daily (hour), homework duration daily (hour), times of vegetable intake weekly, times of fruit intake weekly, times of fast food consumption weekly (P<0.05) (Table 2).

**Table 2:** Univariable analysis of overweight/obesity prevalence among participants in Shandong Province, 2023.

Independent variables	Normal weight(%)	Overweight/obesity(%)	χ^2^	*P*
Physical activity duration daily (hour)			407.106	0.000
< 1	36,518(77.68)	10,493(22.32)		
1 ∼ 1.5	23,173(81.84)	5,143(18.16)		
> 1.5	18,439(83.71)	3,589(16.29)		
Sleep duration daily (hour)			7.629	0.022
< 6	5,721(79.16)	1,506(20.84)		
6 ∼ 8	49,858(80.22)	12,292(19.78)		
> 8	22,551(80.60)	5,427(19.40)		
Screen duration daily (hour)			7.877	0.019
< 1	51,716(80.49)	12,534(19.51)		
1 ∼ 3	21,329(79.90)	5,366(20.10)		
> 3	5,085(79.33)	1,325(20.67)		
Homework duration daily (hour)			7.329	0.026
< 1	37,293(80.61)	8,972(19.39)		
1 ∼ 3	29,573(79.99)	7,396(20.01)		
> 3	11,264(79.77)	2,857(20.23)		
Times of meat intake weekly			1.130	0.568
< 3	36,754(80.29)	9,025(19.71)		
3 ∼ 4	28,509(80.35)	6,974(19.65)		
> 4	12,867(79.95)	3,226(20.05)		
Times of vegetable intake weekly			6.709	0.035
< 4	6,552(79.31)	1,709(20.69)		
4 **∼** 5	19,410(80.06)	4,835(19.94)		
> 5	52,168(80.45)	12,681(19.55)		
Times of fruit intake weekly			6.708	0.035
< 4	18,171(79.92)	4,565(20.08)		
4 **∼** 5	36,070(80.10)	8,962(19.90)		
> 5	23,889(80.74)	5,698(19.26)		
Times of eggs intake weekly			2.092	0.351
< 4	9,681(79.76)	2,456(20.24)		
4 **∼** 5	47,697(80.32)	11,690(19.68)		
> 5	20,752(80.34)	5,079(19.66)		
Times of milk intake weekly			0.583	0.747
< 4	11,976(80.11)	2,973(19.89)		
4 **∼** 5	48,827(80.23)	12,033(19.77)		
> 5	17,327(80.42)	4,219(19.58)		
Times of fast food consumption weekly			31.955	0.000
< 2	53,620(80.67)	12,850(19.33)		
2 **∼** 3	17,829(79.78)	4,518(20.22)		
> 3	6,681(78.25)	1,857(21.75)		

### 3.3 Multivariable analysis of overweight/obesity rate in adolescents from Shandong Province

Using the results of the students' weight status (0 = normal weight, 1 = overweight/obesity) as the dependent variable, and according to the inclusion and exclusion criteria (P=0.05), a total of 15 independent variables were included in the multivariable logistic regression analysis: sex, age, place of residence, family annual income (yuan), weight status of parents, physical activity of parents, mother’s history of GDM, birth weight, physical activity duration daily (hour), sleep duration daily (hour), screen duration daily (hour), homework duration daily (hour), times of vegetable intake weekly, times of fruit intake weekly, times of fast food consumption weekly. Subsequent to the analytical rigour imparted by the Hosmer-Lemeshow test, the logistic regression model exhibited a commendable degree of fit (P>0.05). Several determinants had been identified that decreased the odds of overweight/obesity. Females had demonstrated reduced odds (OR=0.902, 95 %CI: 0.874 ∼ 0.931) compared with males. When age groups were compared, the odds for 14-year-olds were lower (OR=0.940, 95 %CI: 0.898 ∼ 0.985) than for 12-year-olds, while 15-year-olds also presented decreased odds (OR=0.918, 95 %CI: 0.878 ∼ 0.961) compared with 12-year-olds. Adolescents with one active parent had decreased odds (OR=0.917, 95 %CI: 0.883 ∼ 0.952) compared with those with both parents being inactive. Adolescents with both parents actively engaging in physical activities presented even lower odds (OR=0.762, 95 %CI: 0.730 ∼ 0.795) compared with those whose parents were both inactive. Engaging in 1 to 1.5 h of daily physical activity led to decreased odds (OR=0.773, 95 %CI: 0.744 ∼ 0.802) compared with those active for under an hour, and dedicating more than 1.5 h further reduced these odds (OR=0.679, 95 %CI: 0.651 ∼ 0.708) compared with the same reference group. Sleep durations of 6 to 8 h (OR=0.933, 95 % CI: 0.878 ∼ 0.991) and over 8 h (OR=0.908, 95 %CI: 0.852 ∼ 0.969) both resulted in decreased odds compared with those sleeping less than 6 h. Consuming vegetable over 5 times weekly decreased odds (OR=0.934, 95 %CI: 0.882 ∼ 0.989) compared with consumption frequencies of less than 4 times a week, and similarly, fruit consumption more than 5 times weekly resulted in reduced odds (OR=0.951, 95 %CI: 0.910 ∼ 0.993) compared with less frequent intakes. On the other hand, some factors increased the odds of overweight/obesity. Adolescents from urban areas had higher odds (OR=1.055, 95 %CI: 1.020 ∼ 1.090) compared with their rural counterparts. A family income of more than 300,000 yuan resulted in higher odds (OR=1.084, 95 %CI: 1.019 ∼ 1.152) compared with families earning 100,000 yuan or less. Having one overweight/obese parent increased the odds (OR=1.257, 95 %CI:1.211 ∼ 1.305) compared with families where neither parent was overweight/obese, and this risk further increased when both parents were overweight/obese (OR=1.468, 95 %CI:1.379 ∼ 1.562) compared with the same reference group. A maternal history of GDM increased odds (OR=1.520, 95 %CI:1.377 ∼ 1.678) compared with mothers without such history. High birth weight in infants resulted in increased odds (OR=1.159, 95 %CI: 1.086 ∼ 1.238) compared with infants of normal weight. Screen duration of 1 to 3 h (OR=1.040, 95 %CI: 1.003 ∼ 1.078) and more than 3 h (OR=1.080, 95 %CI: 1.014 ∼ 1.152) both resulted in higher odds compared with less than an hour of screen duration. Engaging in 1 to 3 h of homework led to higher odds (OR=1.036, 95 %CI: 1.001 ∼ 1.073) compared with less than an hour, and dedicating more than 3 h raised the odds further (OR=1.052, 95 %CI: 1.003 ∼ 1.103) compared with the same group. Consuming fast food 2 ∼ 3 times weekly increased the odds (OR=1.058, 95 %CI: 1.018 ∼ 1.099) compared with less than 2 times a week, and consumption frequencies of more than 3 times a week led to even higher odds (OR=1.159, 95 %CI: 1.096 ∼ 1.224) compared with the same reference frequency (Table 3.).

**Table 3:** Multivariable analysis of overweight/obesity prevalence among participants in Shandong Province, 2023.

Independent variables	*OR*(95 %*CI*)	*P*
Sex		
Male	1	
Female	0.902(0.874 ∼ 0.931)	0.000
Age		
12	1	
13	0.959(0.915 ∼ 1.004)	0.076
14	0.940(0.898 ∼ 0.985)	0.010
15	0.918(0.878 ∼ 0.961)	0.000
Place of residence		
Rural	1	
Urban	1.055(1.020 ∼ 1.090)	0.002
Family annual income (yuan)		
≤ 100,000	1	
100,001 **∼** 200,000	1.007(0.972 ∼ 1.045)	0.690
200,001 **∼** 300,000	1.045(0.993 ∼ 1.100)	0.093
≥ 300,000	1.084(1.019 ∼ 1.152)	0.010
Weight status of parents		
Neither parent overweight/obesity	1	
One parent overweight/obesity	1.257(1.211 ∼ 1.305)	0.000
Both parents overweight/obesity	1.468(1.379 ∼ 1.562)	0.000
Physical activity of parents		
Neither parent active	1	
One parent active	0.917(0.883 ∼ 0.952)	0.000
Both parents active	0.762(0.730 ∼ 0.795)	0.000
Mother’s history of GDM		
No	1	
Yes	1.520(1.377 ∼ 1.678)	0.000
Birth weight		
Normal birth weight	1	
Low birth weight	0.998(0.939 ∼ 1.060)	0.937
High birth weight	1.159(1.086 ∼ 1.238)	0.000
Physical activity duration daily (hour)		
< 1	1	
1 ∼ 1.5	0.773(0.744 ∼ 0.802)	0.000
> 1.5	0.679(0.651 ∼ 0.708)	0.000
Sleep duration daily (hour)		
< 6	1	
6 ∼ 8	0.933(0.878 ∼ 0.991)	0.025
> 8	0.908(0.852 ∼ 0.969)	0.003
Screen duration daily (hour)		
< 1	1	
1 ∼ 3	1.040(1.003 ∼ 1.078)	0.033
> 3	1.080(1.014 ∼ 1.152)	0.018
Homework duration daily (hour)		
< 1	1	
1 ∼ 3	1.036(1.001 ∼ 1.073)	0.044
> 3	1.052(1.003 ∼ 1.103)	0.037
Times of vegetable intake weekly		
< 4	1	
4 **∼** 5	0.955(0.897 ∼ 1.016)	0.143
> 5	0.934(0.882 ∼ 0.989)	0.019
Times of fruit intake weekly		
< 4	1	
4 **∼** 5	0.989(0.950 ∼ 1.030)	0.598
> 5	0.951(0.910 ∼ 0.993)	0.023
Times of fast food consumption weekly		
< 2	1	
2 **∼** 3	1.058(1.018 ∼ 1.099)	0.004
> 3	1.159(1.096 ∼ 1.224)	0.000

## 4 Discussion

In the Shandong Province, a significant segment of adolescents aged 12 to 15 faced the challenge of overweight/obesity, reflecting broader public health concerns. During this pivotal phase of adolescent growth, many are contending with issues related to weight, potentially impacting their long-term health.

Our study revealed notable sex disparities, with a marginally higher prevalence of overweight/obesity in boys compared to girls. This observation aligned with similar findings from other research, which also reported a higher rate of overweight/obesity in boys than in girls (Jiang et al., 2023). This discrepancy may illuminate underlying sex-specific physiological changes during adolescence and divergent lifestyle choices. As adolescents navigate through puberty, they experience marked hormonal shifts, metabolic transformations, and structural body changes (Saenger, 2003, Kelsey and Zeitler, 2016). In boys, a surge in androgens catalyzes accelerated growth and developmental processes. Meanwhile, girls encounter intensified societal and cultural pressures regarding body image and appearance (Choi and Lim, 2018, Daniels et al., 2020), which might make them more vigilant about their physical appearance. This phenomenon underscores the need to consider sex-specific factors in addressing adolescent overweight/obesity.

In the present study, age was identified as a key determinant in the prevalence of overweight/obesity among adolescents. Our findings indicate that the highest rate of overweight/obesity was observed in 12-year-olds, with a noticeable decline by the age of 15. This pattern was consistent with existing literature, which demonstrated a decreasing trend in overweight/obesity rates with advancing age in adolescents (Duncan et al., 2011). The observed shift could be attributed to heightened self-awareness regarding appearance and physique, typically occurring during puberty (Barkhordari-Sharifabad et al., 2020).

The study identified residence as an influential factor, with urban adolescents exhibiting a higher prevalence of overweight/obesity than their rural counterparts. Additionally, family annual income impacted the overweight/obesity rate, with a higher incidence observed in adolescents from more affluent households. Similar study has indicated that higher socioeconomic status contributed to adolescent overweight/obesity (Tang et al., 2010). In many developing countries, overweight/obesity is often seen as a sign of family affluence. These countries have experienced improved socioeconomic conditions in recent decades, typically associated with better health outcomes and increased resource availability. However, in lower to middle-income countries, higher socioeconomic status might lead to a more automated, less physically active lifestyle, further contributing to overweight/obesity (Mistry and Puthussery, 2015).

Our study found that the weight status of parents was a significant factor contributing to adolescent overweight/obesity. This observation aligned with findings from analogous study, which suggested an increased propensity for offspring to develop overweight/ obesity conditions when either or both parents exhibit similar weight statuses (Næss et al., 2016). Genetics play a pivotal role in adolescent weight issues, including obesity (de Roo et al., 2023, Abdussalam et al., 2017). Further investigation into this area has identified specific genes associated with an increased risk of overweight/obesity (Tang et al., 2014, Mao and Huang, 2015, Ren et al., 2019). If an adolescent's parents or other family members have previously struggled with overweight/obesity issue, it suggests a higher likelihood for these youths to inherit these predisposing genes. This doesn't necessarily mean that adolescents with these genes are destined to be overweight/obese, but it certainly heightens their risk.

This study underscored the pivotal role of parental physical activity patterns in shaping the weight status of adolescents. Supporting literature indicated that the synergistic effect of parents engaging in organized leisure-time physical activities, served as an efficacious strategy for preventing excessive body weight in their offspring (Sigmund et al., 2020). The family has a significant impact on a child's growth (Zielińska et al., 2021). The formation of a behavioral habit is often influenced by the education and environment received in childhood. These habits, akin to innate traits, can accompany a person throughout their life.

This research delineated the substantial influence of maternal history of GDM and birth weight on the predisposition to overweight/obesity in adolescents. Corresponding studies have established a link between a mother's history of GDM (Andegiorgish et al., 2012) and high birth weight (Zou et al., 2019) with the incidence of overweight/obesity in adolescents. When a mother suffers from GDM, her blood sugar levels can rise, leading to an increased transfer of glucose to the fetus via the placenta. This excessive glucose intake might represent a form of overnutrition in utero (Barbour, 2019), resulting in a higher birth weight for the newborns (Yang et al., 2018). This early nutritional excess could potentially establish a higher weight set point, elevating the risk of overweight/obesity during adolescent years (Gillman et al., 2003, Yu et al., 2011). Concurrently, due to the elevated maternal blood sugar, the fetus might develop insulin resistance (Boerschmann et al., 2010). This early onset insulin resistance might persist as the child grows, further escalating their risk of obesity (Kong et al., 2008). Additional study have underscored a strong association between gut microbiota and body weight (Karvonen et al., 2019), suggesting that GDM during pregnancy might increase a child's obesity risk by influencing gut microbiota composition (Zhu et al., 2022).

The finding of this study underscored the role of daily physical activity duration as a key factor in mitigating the risk of overweight/obesity among adolescents. Aligning with similar research, it has been observed that adolescents who regularly engaged in physical exercise were less likely to be overweight/obese (Croezen et al., 2009). Aerobic exercises assist in burning off excess calories (Ma, 2022), preventing their conversion into fat storage. By enhancing their daily activity levels, adolescents can increase their daily caloric expenditure, thereby balancing the calories consumed with those expended. Moreover, consistent anaerobic exercises can lead to an increase in muscle mass (Ozaki et al., 2022). Muscles, in contrast to fat, burn more calories at rest. This implies that the greater the muscle mass, the higher the basal metabolic rate of adolescents (Hsu et al., 2013), allowing them to burn more calories even when at rest. Additionally, physical exercises enhance the body's insulin response, thereby improving insulin sensitivity (Kuhl et al., 2008). Engaging in physical activity bolsters tissue sensitivity to insulin, facilitating optimal glucose metabolism.

This study identified daily sleep duration as a significant factor influencing the incidence of overweight/obesity in adolescents. Complementary research corroborated this finding, indicating that insufficient sleep duration was associated with an increased likelihood of overweight /obesity among adolescents (Gong et al., 2018). Sleep plays a pivotal role in regulating hormones related to appetite. Insufficient sleep may result in reduced levels of leptin (the satiety hormone) and elevated levels of ghrelin (the hunger hormone) (Schmid et al., 2008), leading to increased appetite and, consequently, excessive caloric intake. Furthermore, a lack of adequate sleep can disrupt the body's internal circadian rhythm (Davies et al., 2014), interfering with normal physiological processes, especially the metabolism of sugars (de Souza et al., 2017) and fats (Wang et al., 2018). This disruption can lower the basal metabolic rate (Benedict et al., 2011), thereby elevating the risk of being overweight/obese.

The current study revealed that sedentary behaviors, including prolonged daily screen time and extensive duration spent on homework, were contributory factors to the prevalence of overweight/obesity among adolescents. Parallel study has demonstrated a clear correlation, showing that prolonged sedentary periods were associated with an elevated risk of overweight/obesity in adolescents (Thibault et al., 2010). Primarily, prolonged sitting may lead to a decrease in adolescent bone density (Gabel et al., 2017) and muscle atrophy (Gianoudis et al., 2015), subsequently lowering the basal metabolic rate. Additionally, a sedentary lifestyle typically translates to reduced energy expenditure. If caloric intake remains unchanged in the face of this decreased activity, it can result in weight gain, subsequently heightening the risk of becoming overweight/obese (Cecchini et al., 2010).

This investigation identified that the weekly consumption patterns of vegetable and fruit significantly influenced the weight status of adolescents. Supporting evidence from parallel study indicated that increased intake of vegetable and fruit was associated with a reduced likelihood of overweight or obesity in adolescents (Menon et al., 2019). Vegetable and fruit are good sources of vitamins, minerals (Szpyrka et al., 2015). Nutrients that are crucial for the body's proper functioning and metabolism. Insufficient intake could lead to a slowed metabolism. Being low in calories (Allan and Allan, 2013) and high in fiber (Storey and Anderson, 2014), vegetable and fruit can help individuals feel satiated with fewer calories (Rolls et al., 2004). The fiber content in them plays a vital role in gut health (Wang et al., 2022), and a decrease in vegetable and fruit consumption might adversely impact digestive functions.

The study revealed that the frequency of fast food consumption among adolescents was a critical factor contributing to the prevalence of overweight/obesity in this age group. Correspondingly, similar research indicated a heightened probability of overweight/obesity in adolescents who frequently partook in fast food (Jakobsen et al., 2023). Fast food is typically nutritionally imbalanced, packed with excessive calories, sugars, salts, and fats, yet lacking in essential proteins, vitamins, minerals, and fibers (Khongrangjem et al., 2018). Excessive reliance on fast food can lead to nutritional deficiencies, weight gain, and consequently, overweight/obesity among adolescents.

## 5 Conclusion

The multidimensional nature of overweight/obesity in adolescents required a comprehensive understanding of its various determinants. Addressing these factors holistically could guide targeted interventions for promoting healthier lifestyles and mitigating the overweight/obesity epidemic among the youth of Shandong Province. Future initiatives should prioritize a multi-pronged approach, encompassing genetic, behavioral, environmental, and societal factors, to effectively combat and reduce the prevalence of overweight/obesity in this age group.

However, while the insights drawn from this study were illuminating, it's crucial to interpret them in light of certain limitations.

Selection bias: While stratified random cluster sampling was robust, there was a possibility that students in the selected schools might not be entirely representative of all students in Shandong Province. There might be inherent differences between schools that were sampled and those that were not.
Measurement errors: The reliability and accuracy of self-reported data, such as that from questionnaires, were often questionable. There might be recall bias, where students might not remember certain activities accurately. Social desirability bias could also influence students to provide responses they deem socially acceptable, rather than what was truthful.
Cross-sectional design: Given the cross-sectional nature of the study, it only provided a snapshot of a single point in time. This design limited our ability to infer causation or to observe changes or trends over time.
Statistical method: Due to the failure of our initial multicategorical approach (Normal weight, Overweight, Obesity) to pass the parallel line test (P<0.05), we simplified our analysis to a binary logistic regression, combining Overweight and obesity into one category. This shift, while necessary, may limit the granularity of our findings.
Geographic limitation: The study focused on Shandong Province and, while it strove to be comprehensive for that region, the findings might not be generalizable to other provinces or countries with different cultural, economic, or environmental conditions.

Considering these limitations could help in understanding the broader context of the study results and where caution might be needed in interpreting and generalizing the findings.

## 6 Funding statement

This research was funded by the Scientific Developmental Foundation Project of Dongying (DJB2023016).

## CRediT authorship contribution statement

**Zhihao Huang:** Writing – review & editing, Writing – original draft, Supervision, Software, Project administration, Methodology, Formal analysis, Conceptualization. **Zhiqi Tian:** Writing – review & editing, Writing – original draft, Visualization, Supervision, Investigation, Formal analysis, Conceptualization. **Jian Cui:** Conceptualization, Visualization, Writing – review & editing. **Guan Wang:** Writing – review & editing, Methodology. **Jiyan Chen:** Formal analysis, Writing – review & editing.

## Declaration of competing interest

The authors declare that they have no known competing financial interests or personal relationships that could have appeared to influence the work reported in this paper.

## Data Availability

The data used during the current study are included in this published article and its supplementary information files.

## References

[b0005] Abdussalam A., Elshenawy O.H., Bin Jardan Y.A., El-Kadi A.O.S., Brocks D.R. (2017). The obesogenic potency of various high-caloric diet compositions in male rats, and their effects on expression of liver and kidney proteins involved in drug elimination. J. Pharm. Sci..

[b0010] Afshin A., Forouzanfar M.H., Reitsma M.B., Sur P., Estep K., Lee A., Marczak L., Mokdad A.H., Moradi-Lakeh M., Naghavi M., Salama J.S., Vos T., Abate K.H., Abbafati C., Ahmed M.B., Al-Aly Z., Alkerwi A., Al-Raddadi R., Amare A.T., Amberbir A., Amegah A.K., Amini E., Amrock S.M., Anjana R.M., Ärnlöv J., Asayesh H., Banerjee A., Barac A., Baye E., Bennett D.A., Beyene A.S., Biadgilign S., Biryukov S., Bjertness E., Boneya D.J., Campos-Nonato I., Carrero J.J., Cecilio P., Cercy K., Ciobanu L.G., Cornaby L., Damtew S.A., Dandona L., Dandona R., Dharmaratne S.D., Duncan B.B., Eshrati B., Esteghamati A., Feigin V.L., Fernandes J.C., Fürst T., Gebrehiwot T.T., Gold A., Gona P.N., Goto A., Habtewold T.D., Hadush K.T., Hafezi-Nejad N., Hay S.I., Horino M., Islami F., Kamal R., Kasaeian A., Katikireddi S.V., Kengne A.P., Kesavachandran C.N., Khader Y.S., Khang Y.H., Khubchandani J., Kim D., Kim Y.J., Kinfu Y., Kosen S., Ku T., Defo B.K., Kumar G.A., Larson H.J., Leinsalu M., Liang X., Lim S.S., Liu P., Lopez A.D., Lozano R., Majeed A., Malekzadeh R., Malta D.C., Mazidi M., McAlinden C., McGarvey S.T., Mengistu D.T., Mensah G.A., Mensink G.B.M., Mezgebe H.B., Mirrakhimov E.M., Mueller U.O., Noubiap J.J., Obermeyer C.M., Ogbo F.A., Owolabi M.O., Patton G.C., Pourmalek F., Qorbani M., Rafay A., Rai R.K., Ranabhat C.L., Reinig N., Safiri S., Salomon J.A., Sanabria J.R., Santos I.S., Sartorius B., Sawhney M., Schmidhuber J., Schutte A.E., Schmidt M.I., Sepanlou S.G., Shamsizadeh M., Sheikhbahaei S., Shin M.J., Shiri R., Shiue I., Roba H.S., Silva D.A.S., Silverberg J.I., Singh J.A., Stranges S., Swaminathan S., Tabarés-Seisdedos R., Tadese F., Tedla B.A., Tegegne B.S., Terkawi A.S., Thakur J.S., Tonelli M., Topor-Madry R., Tyrovolas S., Ukwaja K.N., Uthman O.A., Vaezghasemi M., Vasankari T., Vlassov V.V., Vollset S.E., Weiderpass E., Werdecker A., Wesana J., Westerman R., Yano Y., Yonga G., Zaidi Z., Zenebe Z.M., Zipkin B., Murray C.J.L. (2017). Health effects of overweight and obesity in 195 countries over 25 years. N. Engl. J. Med..

[b0015] Allan K., Allan J.L. (2013). An obesogenic bias in women's spatial memory for high calorie snack food. Appetite.

[b0020] Andegiorgish A.K., Wang J., Zhang X., Liu X., Zhu H. (2012). Prevalence of overweight, obesity, and associated risk factors among school children and adolescents in Tianjin. China. Eur. J. Pediatr..

[b0025] Bagnall A.M., Radley D., Jones R., Gately P., Nobles J., Van Dijk M., Blackshaw J., Montel S., Sahota P. (2019). Whole systems approaches to obesity and other complex public health challenges: a systematic review. BMC Public Health.

[b0030] Barbour L.A. (2019). Metabolic culprits in obese pregnancies and gestational diabetes mellitus: big Babies, big twists, big picture. Diabetes Care.

[b0035] Barkhordari-Sharifabad M., Vaziri-Yazdi S., Barkhordari-Sharifabad M. (2020). The effect of teaching puberty health concepts on the basis of a health belief model for improving perceived body image of female adolescents: a quasi-experimental study. BMC Public Health.

[b0040] Benedict C., Hallschmid M., Lassen A., Mahnke C., Schultes B., Schiöth H.B., Born J., Lange T. (2011). Acute sleep deprivation reduces energy expenditure in healthy men. Am. J. Clin. Nutr..

[b0045] Boerschmann H., Pflüger M., Henneberger L., Ziegler A.G., Hummel S. (2010). Prevalence and predictors of overweight and insulin resistance in offspring of mothers with gestational diabetes mellitus. Diabetes Care.

[b0050] Bridger T. (2009). Childhood obesity and cardiovascular disease. Paediatr. Child Health.

[b0055] Bzovsky S., Phillips M.R., Guymer R.H., Wykoff C.C., Thabane L., Bhandari M., Chaudhary V. (2022). The clinician's guide to interpreting a regression analysis. Eye.

[b0060] Cecchini M., Sassi F., Lauer J.A., Lee Y.Y., Guajardo-Barron V., Chisholm D. (2010). Tackling of unhealthy diets, physical inactivity, and obesity: health effects and cost-effectiveness. Lancet.

[b0065] Chao A.M., Wadden T.A., Berkowitz R.I. (2019). Obesity in adolescents with psychiatric disorders. Curr. Psychiatry Rep..

[b0070] Choi, H.G., Lim, H., 2018. Association between BMI for obesity and distress about appearance in Korean adolescents. J. Korean Med. Sci. 33 (20), e150. doi:10.3346/jkms.2018.33.e150. 10.3346/jkms.2018.33.e150 PMC5944214 29760607

[b0075] Clemmensen C., Petersen M.B., Sørensen T.I.A. (2020). Will the COVID-19 pandemic worsen the obesity epidemic?. Nat. Rev. Endocrinol..

[b0080] Cole T.J., Bellizzi M.C., Flegal K.M., Dietz W.H. (2000). Establishing a standard definition for child overweight and obesity worldwide: international survey. BMJ.

[b0085] Croezen S., Visscher T.L., Ter Bogt N.C., Veling M.L., Haveman-Nies A. (2009). Skipping breakfast, alcohol consumption and physical inactivity as risk factors for overweight and obesity in adolescents: results of the E-MOVO project. Eur. J. Clin. Nutr..

[b0090] Daniels E.A., Zurbriggen E.L., Monique Ward L. (2020). Becoming an object: a review of self-objectification in girls. Body Image.

[b0095] Davies S.K., Ang J.E., Revell V.L., Holmes B., Mann A., Robertson F.P., Cui N., Middleton B., Ackermann K., Kayser M., Thumser A.E., Raynaud F.I., Skene D.J. (2014). Effect of sleep deprivation on the human metabolome. Proc. Natl. Acad. Sci. U. S. A..

[b0100] de Roo M., Hartman C., Veenstra R., Nolte I.M., Meier K., Vrijen C., Kretschmer T. (2023). Gene-environment interplay in the development of overweight. J. Adolesc. Health.

[b0105] de Souza J.F.T., Dáttilo M., de Mello M.T., Tufik S., Antunes H.K.M. (2017). High-intensity interval training attenuates insulin resistance induced by sleep deprivation in healthy males. Front. Physiol..

[b0110] Dong Y., Lau P.W.C., Dong B., Zou Z., Yang Y., Wen B., Ma Y., Hu P., Song Y., Ma J., Sawyer S.M., Patton G.C. (2019). Trends in physical fitness, growth, and nutritional status of Chinese children and adolescents: a retrospective analysis of 1.5 million students from six successive national surveys between 1985 and 2014. Lancet Child Adolesc. Health.

[b0115] Dong Y., Ma J., Song Y., Ma Y., Dong B., Zou Z., Prochaska J.J. (2018). Secular trends in blood pressure and overweight and obesity in Chinese boys and girls aged 7 to 17 years from 1995 to 2014. Hypertension.

[b0120] Duncan S., Duncan E.K., Fernandes R.A., Buonani C., Bastos K.D., Segatto A.F., Codogno J.S., Gomes I.C., Freitas I.F. (2011). Modifiable risk factors for overweight and obesity in children and adolescents from São Paulo. Brazil. BMC Public Health.

[b0125] Fang X., Henao-Mejia J., Henrickson S.E. (2020). Obesity and immune status in children. Curr. Opin. Pediatr..

[b0130] Frayling T.M., Timpson N.J., Weedon M.N., Zeggini E., Freathy R.M., Lindgren C.M., Perry J.R., Elliott K.S., Lango H., Rayner N.W., Shields B., Harries L.W., Barrett J.C., Ellard S., Groves C.J., Knight B., Patch A.M., Ness A.R., Ebrahim S., Lawlor D.A., Ring S.M., Ben-Shlomo Y., Jarvelin M.R., Sovio U., Bennett A.J., Melzer D., Ferrucci L., Loos R.J., Barroso I., Wareham N.J., Karpe F., Owen K.R., Cardon L.R., Walker M., Hitman G.A., Palmer C.N., Doney A.S., Morris A.D., Smith G.D., Hattersley A.T., McCarthy M.I. (2007). A common variant in the FTO gene is associated with body mass index and predisposes to childhood and adult obesity. Science.

[b0135] Gabel L., Macdonald H.M., Nettlefold L., McKay H.A. (2017). Physical activity, sedentary time, and bone strength from childhood to early adulthood: a mixed longitudinal HR-pQCT study. J. Bone Miner. Res..

[b0140] Gianoudis J., Bailey C.A., Daly R.M. (2015). Associations between sedentary behaviour and body composition, muscle function and sarcopenia in community-dwelling older adults. Osteoporos. Int..

[b0145] Gillman M.W., Rifas-Shiman S., Berkey C.S., Field A.E., Colditz G.A. (2003). Maternal gestational diabetes, birth weight, and adolescent obesity. Pediatrics.

[b0150] Gong Q.H., Li S.X., Li H., Cui J., Xu G.Z. (2018). Insufficient sleep duration and overweight/obesity among adolescents in a Chinese population. Int. J. Environ. Res. Public Health.

[b0155] Green T.L. (2014). Examining the temporal relationships between childhood obesity and asthma. Econ. Hum. Biol..

[b0160] Han J.C., Lawlor D.A., Kimm S.Y. (2010). Childhood obesity. Lancet.

[b0165] He Y., Luo B., Zhao L., Liao S. (2022). Influences of the COVID-19 pandemic on obesity and weight-related behaviors among Chinese children: a multi-center longitudinal study. Nutrients.

[b0170] Hsu W.H., Fan C.H., Lin Z.R., Hsu R.W. (2013). Effect of basal metabolic rate on the bone mineral density in middle to old age women in Taiwan. Maturitas.

[b0175] Hu K., Staiano A.E. (2022). Trends in obesity prevalence among children and adolescents aged 2 to 19 years in the US from 2011 to 2020. JAMA Pediatr..

[b0180] Huang Y.K., Chang Y.C. (2022). Oral health: The first step to sustainable development goal 3. J. Formos. Med. Assoc..

[b0185] Jaacks L.M., Vandevijvere S., Pan A., McGowan C.J., Wallace C., Imamura F., Mozaffarian D., Swinburn B., Ezzati M. (2019). The obesity transition: stages of the global epidemic. Lancet Diabetes Endocrinol..

[b0190] Jakobsen D.D., Brader L., Bruun J.M. (2023). Association between food, beverages and overweight/obesity in children and adolescents-a systematic review and meta-analysis of observational studies. Nutrients.

[b0195] Jansen P.W., Verlinden M., Dommisse-van Berkel A., Mieloo C.L., Raat H., Hofman A., Jaddoe V.W., Verhulst F.C., Jansen W., Tiemeier H. (2014). Teacher and peer reports of overweight and bullying among young primary school children. Pediatrics.

[b0200] Jiang X., Zhao X., Zhou J., Zhang X., Song Y., Zhao L. (2023). The relationship between family function and the incidence of overweight/obesity in children and adolescents in Chengdu city, Sichuan province of China: based on latent profile analysis. BMC Public Health.

[b0205] Karvonen A.M., Sordillo J.E., Gold D.R., Bacharier L.B., O'Connor G.T., Zeiger R.S., Beigelman A., Weiss S.T., Litonjua A.A. (2019). Gut microbiota and overweight in 3-year old children. Int. J. Obes..

[b0210] Kelishadi R. (2007). Childhood overweight, obesity, and the metabolic syndrome in developing countries. Epidemiol. Rev..

[b0215] Kelsey M.M., Zeitler P.S. (2016). Insulin resistance of puberty. Curr. Diab. Rep..

[b0220] Khongrangjem T., Dsouza S.M., Prabhu P., Dhange V.B., Pari V., Ahirwar S.K., Sumit K. (2018). A study to assess the knowledge and practice of fast food consumption among pre-university students in Udupi Taluk, Karnataka. India. Clin. Epid. and Glo. Health.

[b0225] Kong A.P., Choi K.C., Ko G.T., Wong G.W., Ozaki R., So W.Y., Tong P.C., Chan J.C. (2008). Associations of overweight with insulin resistance, beta-cell function and inflammatory markers in Chinese adolescents. Pediatr. Diabetes.

[b0230] Koyanagi A., Veronese N., Vancampfort D., Stickley A., Jackson S.E., Oh H., Shin J.I., Haro J.M., Stubbs B., Smith L. (2020). Association of bullying victimization with overweight and obesity among adolescents from 41 low- and middle-income countries. Pediatr. Obes..

[b0235] Kuhl J., Moritz T., Wagner H., Stenlund H., Lundgren K., Baavenholm P., Efendic S., Norstedt G., Tollet-Egnell P. (2008). Metabolomics as a tool to evaluate exercise-induced improvements in insulin sensitivity. Metabolomics.

[b0240] Kukaswadia A., Craig W., Janssen I., Pickett W. (2011). Obesity as a determinant of two forms of bullying in Ontario youth: a short report. Obes. Facts.

[b0245] Lloyd L.J., Langley-Evans S.C., McMullen S. (2012). Childhood obesity and risk of the adult metabolic syndrome: a systematic review. Int. J. Obes..

[b0250] Long X., Li X.Y., Jiang H., Shen L.D., Zhang L.F., Pu Z., Gao X., Li M. (2023). Impact of the COVID-19 kindergarten closure on overweight and obesity among 3- to 7-year-old children. World J. Pediatr..

[b0255] Ma C. (2022). Design and practice of aerobics teaching design based on data fusion algorithm. Wirel. Commun. Mob. Comput..

[b0260] Mao S., Huang S. (2015). A meta-analysis of the association between angiotensin-converting enzyme insertion/deletion gene polymorphism and the risk of overweight/obesity. J. Renin Angiotensin Aldosterone Syst..

[b0265] Menon S., Philipneri A., Ratnasingham S., Manson H. (2019). The integrated role of multiple healthy weight behaviours on overweight and obesity among adolescents: a cross-sectional study. BMC Public Health.

[b0270] Mistry S.K., Puthussery S. (2015). Risk factors of overweight and obesity in childhood and adolescence in South Asian countries: a systematic review of the evidence. Public Health.

[b0275] Musa T.H., Wei L., Li X.S., Pu Y.P., Wei P.M. (2016). Prevalence of overweight and obesity among students aged 7–22 years in Jiangsu Province, China. Biomed. Environ. Sci..

[b0280] Næss M., Holmen T.L., Langaas M., Bjørngaard J.H., Kvaløy K. (2016). Intergenerational transmission of overweight and obesity from parents to their adolescent offspring - the HUNT study. PLoS One.

[b0285] Ozaki G.A.T., Camargo J.C.S., Garcia T.A., Castoldi R.C., Belangero W.D. (2022). Effect of aerobic and anaerobic training on differentergometers in rat muscle and heart tissues. Acta. Ortop. Bras..

[b0290] Rao W.W., Zong Q.Q., Zhang J.W., An F.R., Jackson T., Ungvari G.S., Xiang Y., Su Y.Y., D'Arcy C., Xiang Y.T. (2020). Obesity increases the risk of depression in children and adolescents: results from a systematic review and meta-analysis. J. Affect. Disord..

[b0295] Ren D., Xu J.H., Bi Y., Zhang Z., Zhang R., Li Y., Hu J., Guo Z., Niu W., Yang F., Li W., Xu Y., He L., Yu T., Wu J., Li X., Du J., He G. (2019). Association study between LEPR, MC4R polymorphisms and overweight/obesity in Chinese Han adolescents. Gene.

[b0300] Rolls B.J., Ello-Martin J.A., Tohill B.C. (2004). What can intervention studies tell us about the relationship between fruit and vegetable consumption and weight management?. Nutr. Rev..

[b0305] Rupp K., McCoy S.M. (2019). Bullying perpetration and victimization among adolescents with overweight and obesity in a nationally representative sample. Child Obes..

[b0310] Saenger P. (2003). Dose effects of growth hormone during puberty. Horm. Res..

[b0315] Schmid S.M., Hallschmid M., Jauch-Chara K., Born J., Schultes B. (2008). A single night of sleep deprivation increases ghrelin levels and feelings of hunger in normal-weight healthy men. J. Sleep Res..

[b0320] Sheinbein D.H., Stein R.I., Hayes J.F., Brown M.L., Balantekin K.N., Conlon R.P.K., Saelens B.E., Perri M.G., Welch R.R., Schechtman K.B., Epstein L.H., Wilfley D.E. (2019). Factors associated with depression and anxiety symptoms among children seeking treatment for obesity: A social-ecological approach. Pediatr. Obes..

[b0325] Sigmund E., Sigmundová D., Badura P. (2020). Excessive body weight of children and adolescents in the spotlight of their parents' overweight and obesity, physical activity, and screen time. Int. J. Public Health.

[b0330] Storey M., Anderson P. (2014). Income and race/ethnicity influence dietary fiber intake and vegetable consumption. Nutr. Res..

[b0335] Szpyrka E., Kurdziel A., Matyaszek A., Podbielska M., Rupar J., Słowik-Borowiec M. (2015). Evaluation of pesticide residues in fruits and vegetables from the region of south-eastern Poland. Food Control.

[b0340] Tang K.H., Nguyen H.H., Dibley M.J., Sibbritt D.W., Phan N.T., Tran T.M. (2010). Factors associated with adolescent overweight/obesity in Ho Chi Minh city. Int. J. Pediatr. Obes..

[b0345] Tang L., Ye H., Hong Q., Chen F., Wang Q., Xu L., Bu S., Liu Q., Ye M., Wang D.W., Mai Y., Duan S. (2014). Meta-analyses between 18 candidate genetic markers and overweight/obesity. Diagn. Pathol..

[b0350] Tham K.W., Abdul Ghani R., Cua S.C., Deerochanawong C., Fojas M., Hocking S., Lee J., Nam T.Q., Pathan F., Saboo B., Soegondo S., Somasundaram N., Yong A.M.L., Ashkenas J., Webster N., Oldfield B. (2023). Obesity in South and Southeast Asia-a new consensus on care and management. Obes. Rev..

[b0355] Thibault H., Contrand B., Saubusse E., Baine M., Maurice-Tison S. (2010). Risk factors for overweight and obesity in French adolescents: physical activity, sedentary behavior and parental characteristics. Nutrition.

[b0360] von Elm E., Altman D.G., Egger M., Pocock S.J., Gøtzsche P.C., Vandenbroucke J.P. (2007). The strengthening the reporting of observational studies in epidemiology (STROBE) statement: guidelines for reporting observational studies. Lancet.

[b0370] Wang H., Huang X., Tan H., Chen X., Chen C., Nie S. (2022). Interaction between dietary fiber and bifidobacteria in promoting intestinal health. Food Chem..

[b0365] Wang L.Y., Chyen D., Lee S., Lowry R. (2008). The association between body mass index in adolescence and obesity in adulthood. J. Adolesc. Health.

[b0375] Wang X., Sparks J.R., Bowyer K.P., Youngstedt S.D. (2018). Influence of sleep restriction on weight loss outcomes associated with caloric restriction. Sleep.

[b0380] Weihrauch-Blüher S., Schwarz P., Klusmann J.H. (2019). Childhood obesity: increased risk for cardiometabolic disease and cancer in adulthood. Metabolism.

[b0385] Wen J., Zhu L., Ji C. (2021). Changes in weight and height among Chinese preschool children during COVID-19 school closures. Int. J. Obes..

[b0390] Widhalm K., Ghods E. (2010). Nonalcoholic fatty liver disease: a challenge for pediatricians. Int. J. Obes..

[b0400] Yang S., Guo B., Ao L., Yang C., Zhang L., Zhou J., Jia P. (2020). Obesity and activity patterns before and during COVID-19 lockdown among youths in China. Clin. Obes..

[b0395] Yang D., Luo C., Feng X., Qi W., Qu S., Zhou Y., Sun L., Wu H. (2022). Changes in obesity and lifestyle behaviours during the COVID-19 pandemic in Chinese adolescents: A longitudinal analysis from 2019 to 2020. Pediatr. Obes..

[b0405] Yang Y., Wang Z., Mo M., Muyiduli X., Wang S., Li M., Jiang S., Wu Y., Shao B., Shen Y., Yu Y. (2018). The association of gestational diabetes mellitus with fetal birth weight. J. Diabetes Complications.

[b0410] Yu Z.B., Han S.P., Zhu G.Z., Zhu C., Wang X.J., Cao X.G., Guo X.R. (2011). Birth weight and subsequent risk of obesity: a systematic review and meta-analysis. Obes. Rev..

[b0415] Zhai X., Ye M., Gu Q., Huang T., Wang K., Chen Z., Fan X. (2022). The relationship between physical fitness and academic performance among Chinese college students. J. Am. Coll. Health.

[b0420] Zhang Y.X., Wang Z.X., Zhao J.S., Chu Z.H. (2016). Prevalence of overweight and obesity among children and adolescents in Shandong, China: urban-rural disparity. J. Trop. Pediatr..

[b0425] Zhu Q., Yang X., Zhang Y., Shan C., Shi Z. (2022). Role of the gut microbiota in the increased infant body mass index induced by gestational diabetes mellitus. mSystems.

[b0430] Zielińska M., Łuszczki E., Dereń K., Bartosiewicz A. (2021). The role of parents in shaping good nutrition habits in children. Pediatr. Med. Rodz..

[b0435] Zou Z., Yang Z., Yang Z., Wang X., Gao D., Dong Y., Ma J., Ma Y. (2019). Association of high birth weight with overweight and obesity in Chinese students aged 6–18 years: a national, cross-sectional study in China. BMJ Open.

