# Supplementary material for: Prevalence of overweight/obesity, and associated factors among adolescents aged 12 ∼ 15 in Shandong Province, China: A cross-sectional study
Source: Prev Med Rep. 2024 Jul 22;45:102831. doi: 10.1016/j.pmedr.2024.102831 (PMC11347837; doi:10.1016/j.pmedr.2024.102831)
Supplement: Supplementary Data 1 [file mmc1.docx]

Supplementary Table 1 Questionnaire

Dear participants,

Welcome to our study, which aims to understand the current status of overweight/obesity and its influencing factors among 12 ~ 15-year-old adolescents in Shandong Province. Please take some time to answer the following questions. Please note that this is a completely anonymous questionnaire, and we will not collect any information that can identify your personal identity. There are no right or wrong answers to the questions. We hope you can respond based on your true feelings and actual circumstances.

(1) Sex

⓪ male ① female

(2) Age (year) ___

(3) Place of residence

⓪Rural ① Urban

(4) Family annual income (yuan)

①≤ 100000 ② 100001 **~** 200000 ③ 200001 **~** 300000 ④ ≥ 300000

(5) Father’s education level

① Junior high school or below ② Senior high school ③ Above senior high school

(6) Mother’s education level

① Junior high school or below ② Senior high school ③ Above senior high school

(7) Weight status of parents

① Neither parent overweight/obesity ② One parent overweight/obesity

③ Both parents overweight/obesity

(8) Physical activity of parents

① Neither parent active ② One parent active

③ Both parents active

(9) Mother’s history of GDM

⓪ No ① Yes

1. Birth weight

① Normal birth weight ② Low birth weight ③ High birth weight

(11) Physical activity duration daily (hour)

① < 1 ② 1 ~ 1.5 ③ > 1.5

(12) Sleep duration daily (hour)

① < 6 ② 6 ~ 8 ③ > 8

(13) Screen duration daily (hour)

① < 1 ② 1 ~ 3 ③ > 3

(14) Homework duration daily (hour)

① < 1 ② 1 ~ 3 ③ > 3

1. Times of meat intake weekly

① < 3 ② 3 ~ 4 ③ > 4

(16) Times of vegetable intake weekly

① < 4 ② 4 ~ 5 ③ > 5

(17) Times of fruit intake weekly

① < 4 ② 4 ~ 5 ③ > 5

(18) Times of eggs intake weekly

① < 4 ② 4 ~ 5 ③ > 5

(19) Times of milk intake weekly

① < 4 ② 4 ~ 5 ③ > 5

1. Times of fast food consumption weekly

① < 2 ② 2 ~ 3 ③ > 3
